# Stabilized voltage source inverter for sensitive loads in nuclear installations

**DOI:** 10.1038/s41598-024-65331-7

**Published:** 2024-07-04

**Authors:** Marwa M. Mousa, Khalid F. A. Hussein, Z. Matter, Hussein Eleissawi

**Affiliations:** 1 Electrical Engineering Department, Faculty of Engineering, Al-Azhar University, Cairo, Egypt; 2 Reactors Department, Nuclear Research Centre, Egyptian Atomic Energy Authority, Inshas, Egypt; 3 Microwave Engineering Department, Electronics Research Institute (ERI), Cairo, Egypt

**Keywords:** Energy science and technology, Engineering

## Abstract

The present paper proposes a novel design of a stabilized single-phase voltage-source inverter with pure sinusoidal output voltage for photovoltaic systems employed for feeding sensitive loads in nuclear installations. Such types of loads require a voltage source with quite stabilized magnitude and frequency and pure sinusoidal shape that is free from higher-order harmonics. The inverter is based on an H-bridge four insulated gate bipolar transistors (IGBTs) with gate drivers and optocoupler isolators. A control system based on a fast DSP unit and a microcontroller is designed for cancelation of the higher-order harmonics to produce a pure sinusoidal output with almost zero total harmonic distortion (THD). The sinusoidal purity, frequency and magnitude stabilization are achieved through pulse width modulation (PWM) controlled by a fast-response feedback system that measures the time wave form of the output voltage applied to the load and then calculates the THD, frequency and amplitude. The sinusoidal shaping is performed with aid of a sinusoidal shaping filter (SSF) to fasten the operation of higher-order harmonic cancellation. The frequency measurement is achieved through a frequency counter whereas the amplitude measurement is carried out through a differential amplifier that amplifies the difference between the output voltage of the inverter (while being applied to the load) and reference voltage that determines the desired amplitude. A prototype of the entire system of the proposed voltage-source inverter is fabricated for experimental evaluation of its performance. It is shown through simulation and experimental work that the proposed control system of the inverter output voltage is able to stabilize both the amplitude and frequency of the voltage applied to the load irrespective of the load impedance. Also, it is shown that the THD of the output voltage is $-57 \text{ dB}$ ($0.00025 \%$), which is almost zero. Moreover, the switching losses are considerably reduced and can be negligible owing to the use of the appropriate IGBTs. The efficiency of the proposed inverter is obtained by simulation taking into account all types of losses. Also, the dependence of the inverter efficiency on the load current is investigated. The average efficiency of the proposed voltage-source inverter is shown to be higher than $97\%$.

## Introduction

Some AC-powered equipment utilized in nuclear installations are sensitive to the instabilities of the amplitude and frequency of the AC power system. The critical functions of such equipment in the nuclear installations may be badly affected by the instabilities caused by fluctuations of the amplitude and/or frequency^1,2^. Also, the higher-order harmonics imposed in the electric power system may be dangerous to such AC-powered equipment in the nuclear installations. It often happens that an additional load is added (in parallel) to the loads being energized at the inverter output resulting in a sudden drop in the output voltage. Also, when an energized load is disconnected from the inverter output, a sudden increase in the amplitude of the output voltage may occur. Furthermore, some loads may inject currents with higher order harmonics that may be harmful to such sensitive loads connected to the output of the same inverter. Nevertheless, the stability of the inverter output voltage is vital to electrical power systems in such nuclear installations. For this reason the safe operation of such critical equipment require high-quality voltage source with highly stabilized amplitude and frequency, pure sinusoidal waveform, and high efficiency. The inverter proposed in the present work is a high-quality single-phase voltage source (220 V, 50 Hz), for supplying the vital equipment in nuclear installations as mentioned above.

A stand-alone inverter obtains the DC power from batteries that can be charged by photo voltaic (PV) arrays or by another available source. Those batteries may have built-in battery chargers that can be used to charge the batteries when an AC source is available. One of the most critical issues of the voltage-source inverter design is the control system that realizes the amplitude and frequency stabilization and the sinusoidal purity of the output waveform^3^.

Recently, a lot of research work has been focused on the stabilization of the inverter output voltage as a crucial requirement of the inverters used for photovoltaic systems. In 2018, Diouri et al. simulated a voltage-source inverter model with varying load and tested it by Simulink. Proportional-Integral-Derivative (PID) controller was for correcting the voltage drop leading to good regulation of the output voltage of the inverter^4^. In 2019, Wang et al. proposed a novel combined centralized/decentralized method to achieve voltage regulation by overcoming the challenge of PV power imbalance. The response of the reactive inverter power to PV power changes is adaptively scheduled to overcome the negative effect on the voltage regulation caused by voltage/reactive power interaction^5^. In 2020, Ersan Kabalc conducted a survey on single-phase grid-connected voltage-source inverters regarding their enhancements in circuit design and mechanisms of control^6^. In 2021, Dhanamjayulu et al. suggested a novel 15-level single-phase voltage-source inverter. This inverter helps generate 15 levels of stepped voltage with low total harmonic distortion (THD) and thereby improving the efficiency and reducing the cost and system complexity^7^. In 2022, Lulbadda et al. presented the operation of a voltage-source inverter in reactive power-injection mode in the absence of solar energy. The main concern was the design of the control system of the inverter. The suggested method enabled the injection of the amount of reactive power needed for output voltage regulation^8^. In 2023, C. Choeung et al. demonstrated a control system based on linear matrix inequality (LMI) for a voltage-source inverter with LC filter^9^.

In the present work, a new design of a stand-alone stabilized voltage-source inverter for energizing sensitive equipment in the nuclear installation. This inverter depends on an H-bridge of four insulated gate bipolar transistors (IGBTs) for voltage inversion. Also, it depends on a robust control system to produce pure sinusoidal voltage with stabilized amplitude and frequency. The present work proposes new methods for frequency stabilization, voltage regulation and reduction of the THD of the inverter output voltage, especially while being applied to the sensitive load loads of the nuclear installations as mentioned above. These methods are combined in a control system of fast response. A sinusoidal shaping filter (SSF), which is an LC filter, is connected to the output of the H-bridge to enable producing a sinusoidal waveform of the inverter output voltage through the processes of the control system. The main components of the proposed control system are a microcontroller, a digital signal processing (DSP) unit, and the feedback circuits. The microcontroller is dedicated for switching the H-bridge to produce a square-wave AC voltage with the required duty cycle. The function of the DSP unit is to handle the feedback signals and to issue the proper commands to the microcontroller. One of the feedback signals is generated by direct conversion of the output voltage to DC signal and then input to an analog-to-digital converter (ADC) for fast amplitude regulation. Another feedback signal is a scaled rectified version of the inverter output voltage. After rectification, this signal is subjected to sampling and input to another ADC. From this signal, the actual waveform of the inverter output voltage can be reconstructed by the DSP unit to measure the amplitude, frequency, and THD. By proper switching of the IGBTs of the H-bridge the and by setting the appropriate value of the duty cycle, the amplitude, frequency and THD of the inverter output voltage can be accurately adjusted irrespective of the load impedance. Moreover, the proposed control system enables the suppression of any higher-order harmonics caused by some types of loads. In the control system of the proposed Voltage-source inverter, the Arduino Uno microcontroller is used for direct switching of the H-bridge and the Espressif ESP32-wroom-32 microcontroller with the Espressif DSP library (ESP-DSP) is used to implement the DSP unit for harmonic analysis of the time waveform of the output voltage. The central unit of the control system is the ESP32 DSP whose commands are issued to the Arduino Uno microcontroller through serial communication.

## Methods and results

### Design requirements of the voltage-source inverter

The inverter is proposed as a single-phase 220 V, 50 Hz, voltage source for vital equipment that play critical roles in nuclear installations. These devices must remain in excellent working condition at all times without interruption. They are very sensitive to any variations of the amplitude and frequency of the power source. Such instabilities of the voltage source may lead to malfunction, temporary, or permanent failure during operation of these equipment thereby resulting in dangerous consequences. For this reason the safe operation of such critical equipment require high-quality voltage source with highly stabilized amplitude and frequency, very low THD, and high efficiency.

### Stability requirements of the voltage source

#### Amplitude stability

The amplitude stability of the required voltage source can be realized by minimizing the change of the amplitude from the desired value by minimizing the following errors.

- The amplitude error (AE) is defined as the percent ratio of the mean amplitude error to the desired amplitude.
- The amplitude fluctuation rate (AFR) is defined as the percent ratio of the rms value of the amplitude variation to the rms value of the voltage

#### Frequency stability

The frequency stability of the required voltage source can be realized by minimizing the frequency deviation from the desired frequency by minimizing the following errors.

- The frequency error (FE), which is defined as the percent ratio of the mean frequency error to the desired frequency.
- The frequency fluctuation rate (FFR), which is defined as the percent ratio of the rms value of the frequency variation to the mean value of the frequency.

#### Sinusoidal purity

The sinusoidal purity of the output voltage is crucial for excellent operational conditions the equipment mentioned above. It can be realized by minimizing the THD.

- The THD is defined as the percent ratio of the square root of the total power of the higher-order harmonics to the power of the fundamental harmonics.

#### High Efficiency

To optimize the operation and minimize the need for battery replacement, the inverter efficiency should be maximized.

- The inverter efficiency ($\eta$) is defined as the percent ratio of the power consumed by the load to the power input to the inverter (i.e. the power output from the battery).

Quantitatively, the required performance metrics of the proposed voltage-source inverter are summarized in Table 1. It should be noted that these quantitative requirements were determined based on analytical and experimental studies carried out by the work team charged with implementing safety and efficiency procedures of the equipment in the previously mentioned nuclear facilities.

**Table 1:** Required performance metrics of the proposed voltage-source inverter.

Quality issue	Condition
Amplitude stability	$AE\le 2\%$
	$$AFR\le 1\%$$
Frequency stability	$FE\le 0.5\%$
	$$FFR\le 0.25\%$$
Sinusoidal purity	$THD\le 1\%$
High efficiency	$\eta \ge 95\%$

### Design of the inverter switching circuit

As mentioned in the introduction, the proposed voltage-source inverter belongs to the category of stand-alone inverters that obtain the DC input from batteries that can be charged with the PV array or by an AC source if present and usable. The design of the proposed voltage-source inverter includes the basic IGBT circuit, the IGBT gate driver and the optocoupler isolator.

#### Basic IGBT circuit for voltage inversion

**Figure 1:**
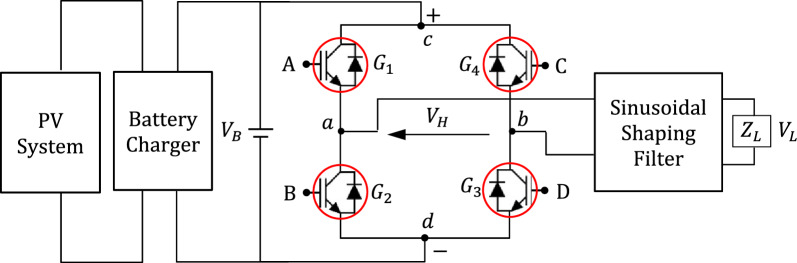
The basic IGBT H-bridge circuit of the proposed inverter.

The basic IGBT H-bridge circuit designed for the proposed voltage-source inverter is presented in Fig. 1. The positive terminal of the battery is connected to the point $c$ of the H-bridge whereas the point $d$ is connected to the negative terminal. The output voltage of the bridge is the voltage between the points $a$ and $b$, ${V}_{H}={V}_{a}-{V}_{b}$. If ${V}_{Aa}>{V}_{Th}$ and ${V}_{Dd}>{V}_{Th}$, whereas ${V}_{Cb}=0$ and ${V}_{Bd}=0$, then the output voltage, ${V}_{H}$ is positive (${V}_{H}\approx +{V}_{B}$). If ${V}_{Cb}>{V}_{Th}$ and ${V}_{Bd}>{V}_{Th}$, whereas ${V}_{Aa}=0$ and ${V}_{Dd}=0$, then the output voltage, ${V}_{H}$ is negative (${V}_{H}\approx -{V}_{B}$). Thus, ${V}_{H}$ is, primarily, a square-wave with zero-mean (i.e. zero DC component). The H-bridge shown in Fig. 1 is controlled through a fast digital controller that is responsible for generating the control signals at A, B, C and D.

#### IGBT gate driver

**Figure 2:**
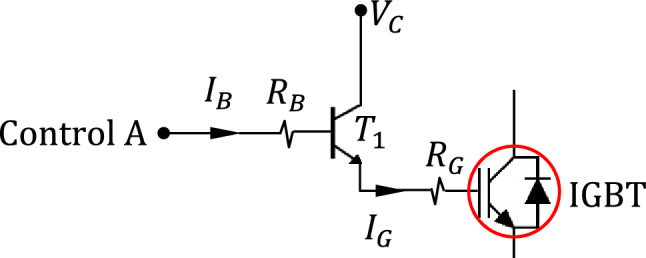
IGBT gate driver without optocoupler isolator.

Bipolar junction transistors (BJTs) are inserted between the digital controller and the H-bridge to play the dual role of protecting the digital controller and amplifying the current supplied to the gates of the IGBTs of the H-bridge. For example, a BJT current buffer is used as shown in Fig. 2. Note that the BJT ${T}_{1}$ is biased to operate in the active region. It can be shown that the base current ${I}_{B}$ drawn from the microcontroller is given as follows.

1 $${I}_{B}=\frac{{V}_{C}-{V}_{\gamma }}{\left(1+\beta \right) {R}_{G}}$$

where ${V}_{\gamma }\approx 0.7 \, \text{V}$, $\beta \approx 300$ is the current amplification for the BJT model 2N3904. If these values are used in (1), one gets ${I}_{B}\approx 0.3 \, \text{mA}$, which is very low current that can be drawn from the digital controller without causing harmful load.

#### Optical isolation of the IGBT circuit

**Figure 3:**
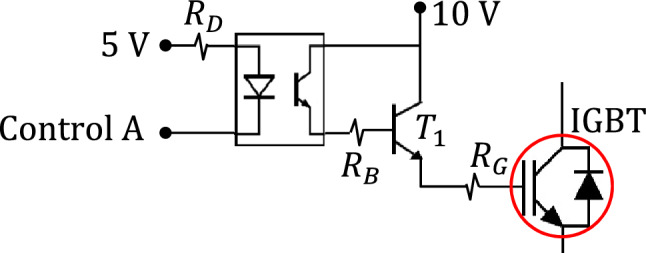
Optocoupler isolator is connected for protection of the control circuit.

For more protection of the digital controller and for more reduction of the current drawn from the digital controller, an optocoupler isolator can be used as shown in Fig. 3. Active-low control logic is used such that the digital controller sinks the current to turn on the IGBT.

#### Complete design of the inverter switching circuit

**Figure 4:**
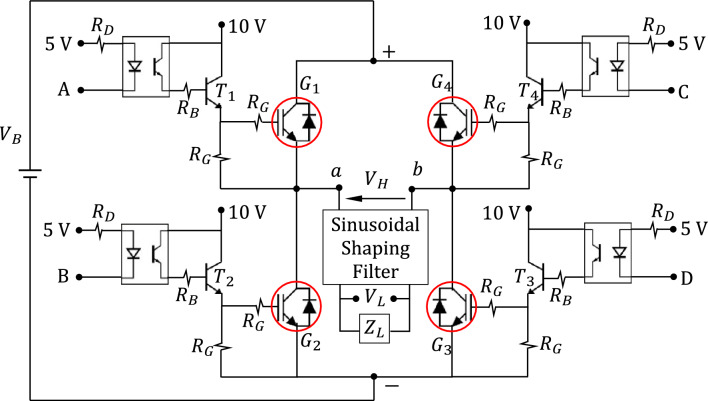
Circuit diagram of the IGBT H-bridge for the proposed voltage-source inverter, the IGBT gate drivers and optoisolators.

The complete circuit diagram of the IGBT H-bridge designed for the proposed voltage-source inverter including the interface circuit between the microcontroller and the H-bridge is presented in Fig. 4. This interface protects the microcontroller from possible high currents that can be drawn from it during operation. The protective interface circuit includes the IGBT gate drivers and the optocoupler isolators as presented in Fig. 4.

### Analysis of the output voltage of the IGBT H-bridge

#### Time waveforms of switching the H-bridge for efficient voltage inversion

**Figure 5:**
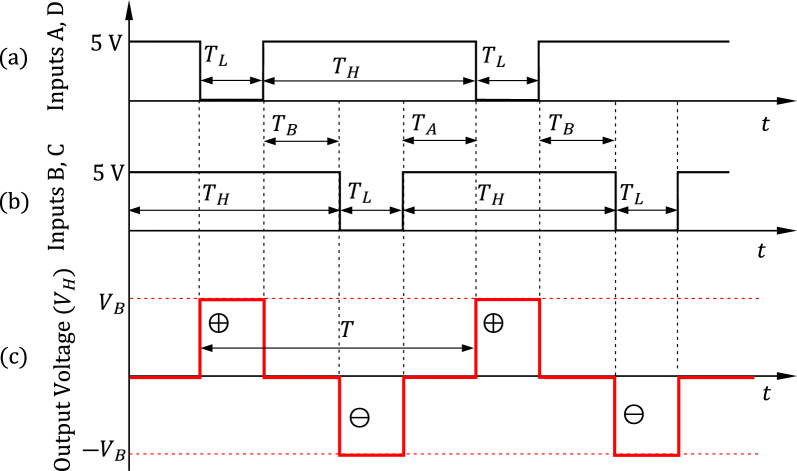
Sketched time waveforms of the IGBT H-bridge input control signals (A, D) and (B, C) and the corresponding inverter output voltage, ${V}_{H}$, at no-load.

The time waveforms of the input control signals A and B of the IGBT H-bridge and the corresponding output voltage, ${V}_{H}$, can be sketched as shown in Fig. 5. When the inputs A, D are logically “Low” whereas the logic inputs B, C are logically “High”, the transistors ${G}_{1}$ and ${G}_{3}$ are saturated and the transistors ${G}_{2}$ and ${G}_{4}$ are off. In this case, the H-bridge output voltage, ${V}_{H}$, is positive and almost equal to the battery voltage, ${V}_{B}$. In a similar way, when the inputs B, C are logically “Low” whereas the inputs A, D are logically “High”, the inverter output voltage, ${V}_{H}$, is almost equal to $-{V}_{B}$. The frequency of the H-bridge output signal, ${f}_{H}$, can be determined as follows.

2 $${f}_{H}=\frac{1}{{T}_{L}+{T}_{H}}$$

Let us define the ratios $R$ and $D$ as follows.

3 $$R=\frac{{T}_{H}}{{T}_{L}}, D=\frac{2{T}_{L}}{{T}_{H}+{T}_{L}}=\frac{2}{R+1}$$

Note that $D$ is the duty cycle of the output square wave of the H-bridge. The root-mean-squared (rms) value of the H-bridge output voltage, ${V}_{H rms}$, can be expressed in terms of the battery voltage and the duty cycle as follows.

4 $${V}_{H rms}={V}_{B}\sqrt{\frac{2}{R+1}}={V}_{B}\sqrt{D}$$

Setting $T={T}_{H}+{T}_{L}=0.02 \, \text{ s}$ results in a square wave with frequency $50\, \text{ Hz}$. In this case, the principal harmonic of the Fourier series expansion will be at $50\, \text{ Hz}$.

#### Total harmonic distortion of the H-bridge output voltage

The time waveform of the output voltage of the H-bridge can be expressed in a Fourier series as follows.

5 $${v}_{H}\left(t\right)={\sum }_{n=1}^{\infty }{a}_{Hn}\mathit{cos}({\omega }_{n}t)={\sum }_{n=1}^{\infty }{a}_{Hn}\mathit{cos}\left(n{\omega }_{r}t\right)$$

where,${\omega }_{r}=2\pi {f}_{r}$ and ${f}_{r}=50\, \text{ Hz}$ is the fundamental harmonic frequency.

Let ${H}_{H}$ be the total harmonic distortion (THD) of the square wave at the H-bridge output; this can be expressed as follows.

6 $${H}_{H}=\frac{1}{{{a}_{H}}_{1}}\sqrt{\sum_{n\ge 2}{a}_{Hn}^{2}}$$

where ${a}_{Hn}$ is the magnitude of the *n*th-order harmonic of the Fourier expansion. As the frequency of the square wave is 50 Hz, the magnitude of the 1st-order harmonic, ${a}_{H1}$, will be much greater than the higher-order harmonics (${a}_{H1}\gg {a}_{Hn}, n\ge 2$). However, the Fourier series of the square wave is slowly convergent as it has very slowly decaying magnitudes and, hence, large number of terms is required for accurate calculation of the series in (5). The rms value of the H-bridge output voltage, ${V}_{H rms}$, can be expressed in terms of the principal harmonic magnitude, ${a}_{H1}$, and the THD as follows.

7 $${V}_{H rms}={{a}_{H}}_{1}\sqrt{\frac{1+{{H}_{H}}^{2}}{2}}$$

Comparing the expression (7) to (4), it can be shown that,

8 $${a}_{H 1}={V}_{B}\sqrt{\frac{2D}{1+{{H}_{H}}^{2}}}$$

The normalized power of the battery voltage is ${V}_{B}^{2}$ (i.e. when applied to one-$\Omega$ resistive load), whereas the normalized power of the 1st-order harmonic is ${a}_{H1}^{2}/2$. Thus, ${H}_{H}$ can be expressed as follows.

9 $${H}_{H}=\sqrt{\frac{D {V}_{B}^{2}- \frac{1}{2}{a}_{H1}^{2}}{\frac{1}{2}{a}_{H1}^{2}}}=\sqrt{\frac{2D {V}_{B}^{2}}{{a}_{H1}^{2}}-1}$$

The last equation means that to calculate the THD of the square wave voltage at the H-bridge output, it is enough to get the magnitude of the first harmonic, ${a}_{H1}$.

### Simulation results for the H-bridge output voltage

**Figure 6:**
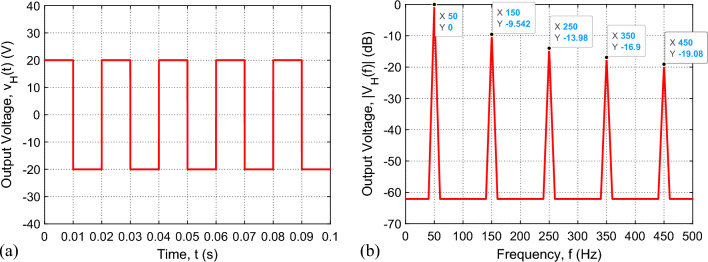
The output voltage of the H-bridge: (**a**) Time waveform, ${v}_{H}\left(t\right)$. (**b**) Normalized frequency spectrum magnitude, $\left|{V}_{H}\left(\omega \right)\right|$. $T=0.02 \, \text{s}$ and duty cycle, $D=1$.

To test the functionality of the IGBT H-bridge, the Proteus simulator is employed and the schematic diagram shown in Fig. 4 is used to monitor the time waveform of the output voltage at the indicated test points $a$ and $b$. It should be noted that the model of the Arduino Uno™ microcontroller is used in the Proteus simulator to generate the control signals (A, B, C, and D shown in Fig. 4). The time waveform of the H-bridge output voltage, ${v}_{H}(t)$, (voltage difference between $a$ and $b$) obtained by simulation, is presented in Fig. 6a. The corresponding spectrum magnitude, $\left|{V}_{H}\left(\omega \right)\right|$, obtained by applying the fast Fourier transform (FFT), is presented in Fig. 6b. It is shown that only the odd-order harmonics exist where the 1st-order harmonic is exactly at 50 Hz and its magnitude is about three times the magnitude of the 3rd-order harmonic, which is the maximum magnitude among those of the higher-order harmonics. The THD calculated using expression (9) gives ${THD}_{H}\approx 50\%$ ($-3 \, \text{ dB}$), which means that the power of the 1st-order harmonic is almost equal to twice the power the of all the other (higher-order) harmonics.

### Experimental measurement of the H-bridge output voltage

**Figure 7:**
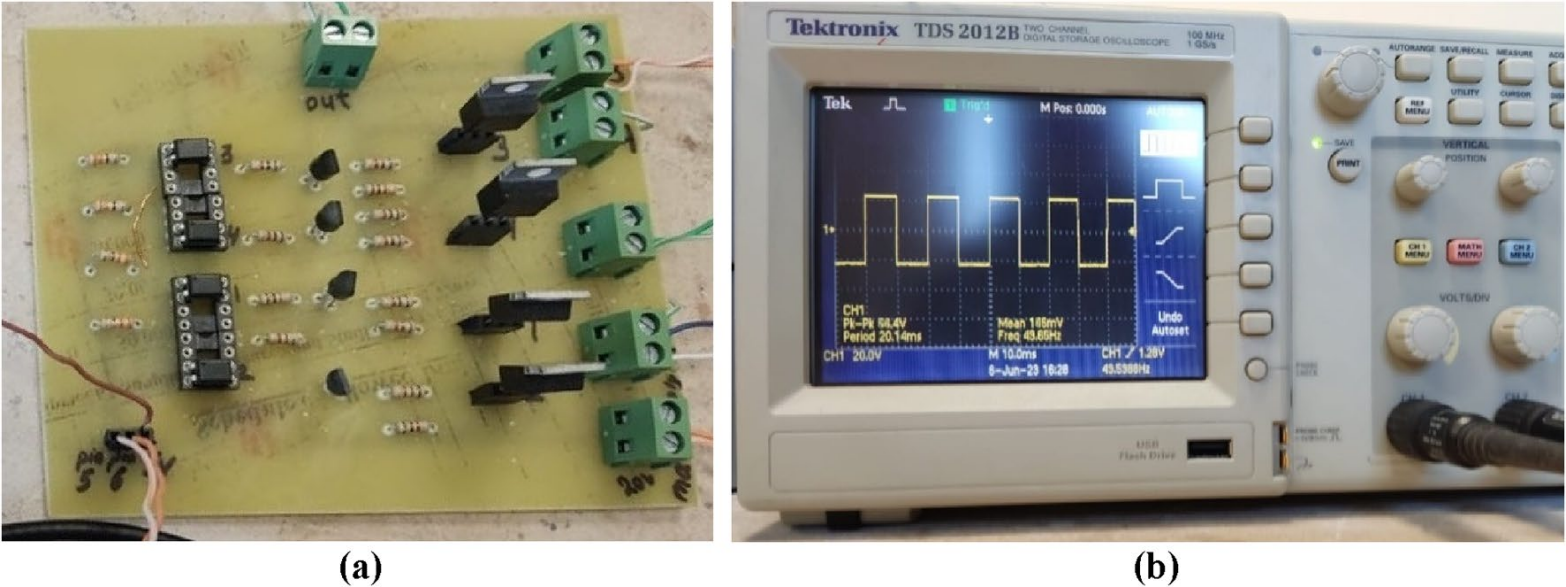
The fabricated IGBT H-bridge circuit produces a square-wave output voltage of frequency 50 Hz when the duty cycle is set to $D=1$. (**a**) Fabricated H-bridge circuit of the schematic shown in Fig. 4. (**b**) The output square wave is displayed by the Tektronix oscilloscope model TDS2012B.

For experimental testing of the switching IGBT H-bridge, the circuit prototype shown in Fig. 7a is fabricated. The AC square-wave voltage at the output of the H-bridge is measured using Tektronix oscilloscope model TDS 2012B as shown in Fig. 7b. This model of the oscilloscope has the capability of spectral analysis through the built-in FFT option.

**Figure 8:**
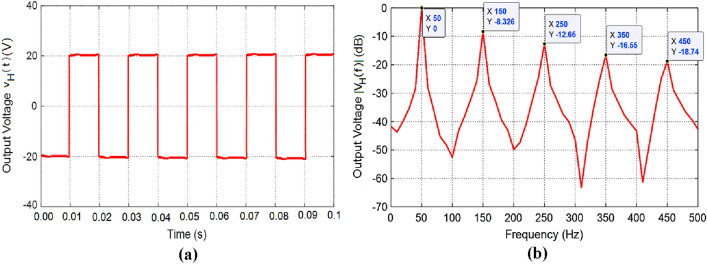
Output voltage of the IGBT H-bridge (without applying the SSF), as measured by Tektronix oscilloscope model TDS2012B for duty cycle $D=1$. (**a**) Time waveform, ${v}_{H}(t)$. (**b**) Normalized spectrum magnitude, $\left|{V}_{H}\left(f\right)\right|$, obtained by the FFT option of the oscilloscope.

The results of experimental measurements of the AC voltage at the output of the fabricated prototype of the IGBT H-bridge are presented in Fig. 8. These experimental results correspond to the simulation results presented in Fig. 6. The AC square-wave voltage at the output of the fabricated H-bridge is measured using Tektronix oscilloscope model TDS 2012B where the measured waveform is presented in Fig. 8a. This oscilloscope has the capability of spectral analysis through the application of the built-in FFT option. The measured spectrum of the of the H-bridge output voltage is presented in Fig. 8b. It is shown that the simulation and measurement results shown in Figs. 6 and 8, respectively, come in good agreement with each other.

### Sinusoidal shaping of the inverter output Voltage

A fast-response control system is designed to achieve the objectives of the proposed voltage-source inverter including voltage regulation, frequency stabilization, and minimization of THD. This control system requires a SSF at the output of the H-bridge. In the present section, an LC filter resonating at the desired frequency, ${f}_{r}$, of the output voltage (usually 50 Hz) is proposed.

#### LC Filter for sinusoidal shaping of the H-bridge output

**Figure 9:**
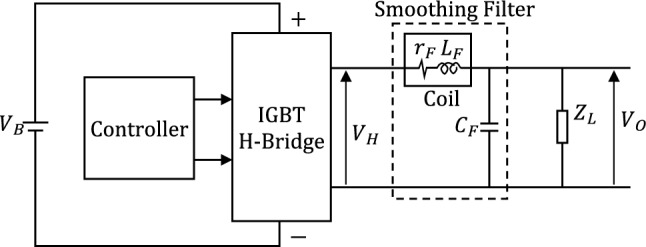
Sinusoidal shaping of the inverter output voltage using an LC filter resonant at $50 \, \text{ Hz}$.

The objective of the voltage-source inverter design is to achieve a pure sinusoidal time waveform of the AC voltage with the desired frequency at the inverter output. Therefore, it is proposed to suppress the higher-order harmonics of the square-wave H-bridge output voltage using the LC-filter as shown in Fig. 9. The proposed LC filter is constructed of a series coil and shunt capacitor inserted between the H-bridge and the load as shown in the figure. By setting the appropriate values of the inductance and capacitance, such LC filter can be proposed for sinusoidal shaping of the square-wave output voltage. The coil has an inductance ${L}_{F}$ and an (unavoidable) internal resistance ${r}_{F}$ that is usually very small for high-quality wire-wound coil. Thus, the resistance ${r}_{F}$ is proportional to the length of the coil wire, i.e. the number of turns. Also, the coil inductance, ${L}_{F}$, is proportional to the number of coil turns as explained in Appendix B. Also, ${r}_{F}$ is proportional to ${L}_{F}$, and one can write the following expression.

10 $${r}_{F}=\alpha {L}_{F}$$

where $\alpha$ is the constant of proportionality (defined in Appendix B) and has the units $\Omega /\text{H}$.

The filter output can be expressed as Fourier series expansions with frequency harmonics that are integer multiples of the fundamental frequency $50 \, \text{ Hz}$.

11 $${v}_{O}\left(t\right)={\sum }_{n=1}^{\infty }{a}_{On}\mathit{cos}\left(n{\omega }_{r}t\right)$$

**Figure 10:**
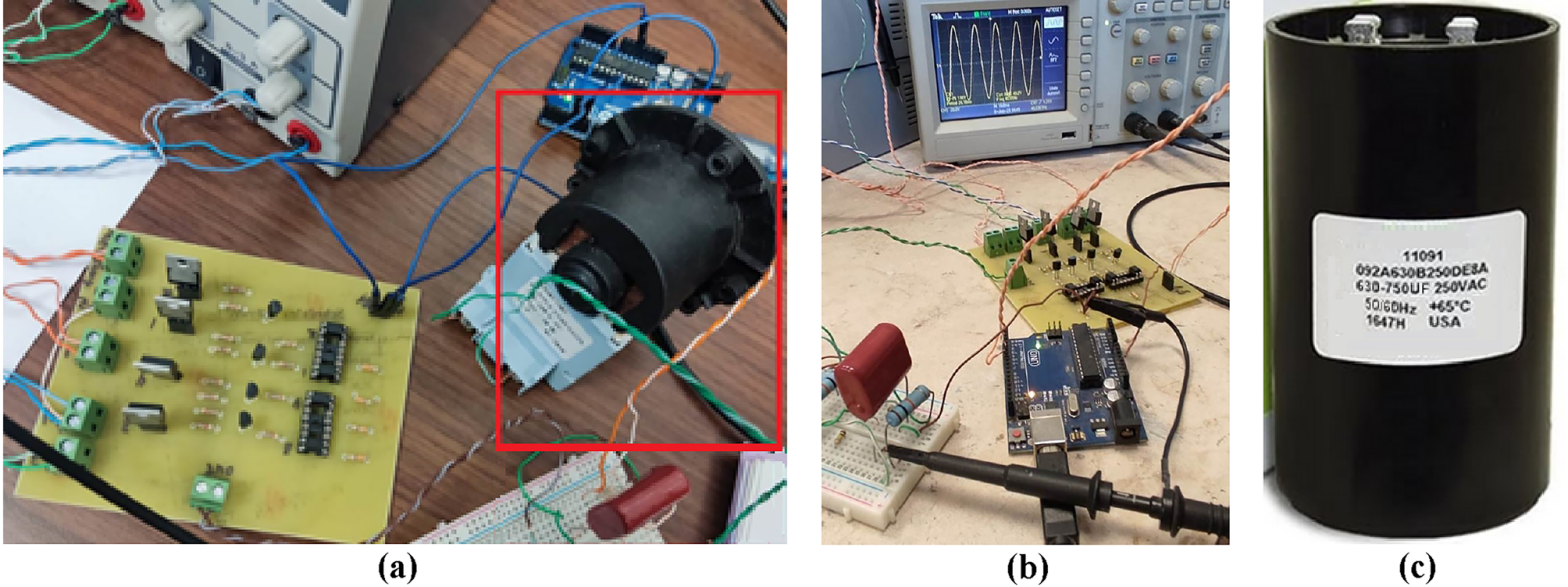
Experimental evaluation of the effect of the SSF. (**a**) The fabricated inverter with the SSF and the controller. The coil of the SSF is bounded by a red frame. (**b**) The time waveform of the voltage at the output of the SSF is displayed by the Tektronix oscilloscope model TDS2012B. (**c**) The $750 \, \upmu \text{F}$, 250 VAC capacitor from BMI (Airstar Supply) manufacturer.

Consider that the output impedance of the H-bridge is much smaller than the input impedance of the SSF while the load is being connected in parallel with the filter capacitor as shown in Fig. 10. Thus, the following expression can be obtained for the transfer function of the filter/load combination, which is the relation between the inverter output voltage, ${V}_{o}(\omega )$, and the output voltage of the H-bridge, ${V}_{H}(\omega )$.

12 $${G}_{F}\left(\omega \right)=\frac{{V}_{o}\left(\omega \right)}{{V}_{H}(\omega )}= \frac{{Z}_{L}}{{Z}_{L}\left(1-{\omega }^{2}{L}_{F}{C}_{F}\right)+{r}_{F}+j\omega \left({{L}_{F}+C}_{F}{r}_{F}{Z}_{L}\right)}$$

where ${Z}_{L}$ is the load impedance connected at the inverter output.

To filter out all the frequency harmonics except for that with $f={f}_{r}$, it is required that ${G}_{F}\left(\omega \right)$ has a sharp maximum at ${f}_{r}$. This can be achieved if ${\omega }_{r}^{2}{L}_{F}{C}_{F}=1$, where ${\omega }_{r}=2\pi {f}_{r}$. This requires that ${C}_{F}$ and ${L}_{F}$ are related to each other through the following expression.

13 $${C}_{F}=\frac{1}{{\omega }_{r}^{2}{L}_{F}}$$

At resonance ($\omega ={\omega }_{r}$), condition (13) is satisfied and expression (12) can be reduced to get the following expression.

14 $${G}_{F}\left(\omega \right)= \frac{{Z}_{L}}{{r}_{F}+j{\omega }_{r}\left({{L}_{F}+C}_{F}{r}_{F}{Z}_{L}\right)}$$

The filter gain, ${\mathcal{G}}_{F}$, is the magnitude of ${G}_{F}\left(\omega \right)$ at resonance.

15 $${\mathcal{G}}_{F}\stackrel{\scriptscriptstyle\text{def}}{=}\left|{G}_{F}\left({\omega }_{r}\right)\right|$$

For well-designed wire-wound coil on MnZn ferrite core (see Appendix B), ${r}_{F}$ is very small and can be neglected. Hence, the filter gain can be expressed as follows.

16 $${\mathcal{G}}_{F}= \frac{\left|{Z}_{L}\right|}{{\omega }_{r}{L}_{F}}$$

This means that decreasing the filter coil inductance, ${L}_{F}$, increases the filter gain and, hence, increases the amplitude of the amplitude of the inverter output voltage. Moreover, since the coil resistance, ${r}_{F}$, is proportional to the coil inductance, ${L}_{F}$, it is recommended to decrease ${L}_{F}$ to reduce the voltage drop across ${r}_{F}$ and to reduce the power dissipated in the SSF.

Since the relation (13) is satisfied, the THD of the output voltage is very small and the output of the filter is almost sinusoidal and can be approximated as follows.

17 $${v}_{O}\left(t\right)\approx {A}_{O}\mathit{sin}({\omega }_{r}t)$$

where ${A}_{O}$ is the amplitude of the sinusoidal voltage applied to the load at the SSF output and can be expressed as follows.

18 $${A}_{O}\approx {{a}_{O}}_{1}=\sqrt{2} \left|{V}_{O}\left({\omega }_{r}\right)\right|$$

Replacing ${V}_{O}\left({\omega }_{r}\right)$ by ${G}_{F}\left({\omega }_{r}\right){ V}_{H}\left({\omega }_{r}\right)$ in (18), the amplitude ${A}_{O}$ can be expressed as follows.

19 $${A}_{O}=\sqrt{2} \left|{G}_{F}\left({\omega }_{r}\right){ V}_{H}\left({\omega }_{r}\right)\right|$$

From the harmonic analysis of the H-bridge output voltage detailed above, it can be shown that, if ${f}_{r}=1/T$, then,

20 $$\left|{V}_{H}\left({\omega }_{r}\right)\right|=\frac{{a}_{1H}}{\sqrt{2}}$$

Substituting from (20) into (19) and making use of (15), the flowing expression is obtained for ${A}_{O}$.

21 $${A}_{O}={\mathcal{G}}_{F}{V}_{B}\sqrt{\frac{2D}{1+{{H}_{H}}^{2}}}$$

Substituting for ${\mathcal{G}}_{F}$ from (16) into (21), gives the following

22 $${A}_{O}= \frac{\left|{Z}_{L}\right|}{{\omega }_{r}{L}_{F}}{V}_{B}\sqrt{\frac{2D}{1+{{H}_{H}}^{2}}}$$

Substituting for ${A}_{0}$ from (22) into (17), the following approximation is obtained.

23 $${v}_{O}\left(t\right)\approx \frac{\left|{Z}_{L}\right|}{{\omega }_{r}{L}_{F}}\sqrt{\frac{2D}{1+{{H}_{H}}^{2}}} {V}_{B}\mathit{sin}({\omega }_{r}t)$$

As mentioned above, for well-designed SSF, ${H}_{H}$ is very small and can be neglected in (21) and (22). By setting $D=1$ and ${H}_{H}=0$ in (22), the following expression is obtained for ${A}_{0}$.

24 $${A}_{O}\approx \sqrt{2} \frac{\left|{Z}_{L}\right|}{{\omega }_{r}{L}_{F}}{V}_{B}, for D=1$$

Substituting for ${A}_{0}$ from (24) into (17), the following approximate expression is obtained for the time waveform of the inverter output voltage, ${v}_{O}\left(t\right)$, when applied to load of arbitrary impedance ${Z}_{L}$.

25 $${v}_{O}\left(t\right)\approx \sqrt{2} \frac{\left|{Z}_{L}\right|}{{\omega }_{r}{L}_{F}}{V}_{B} \mathit{sin}({\omega }_{r}t), for D=1$$

#### Experimental measurement of the voltage at the output of the SSF

For experimental measurement of the inverter output voltage, the SSF is fabricated and connected to the output of the IGBT H-bridge as shown in Fig. 10a. The coil is fabricated as a copper wire of diameter $2 \, \text{ mm}$ wound around a cylindrical MnZn ferrite core of diameter $3 \, \text{ cm}$ and length $1.2 \, \text{ cm}$. The number of turns is 6 to produce coil inductance ${L}_{F}=13.5 \, \text{mH}$ and coil resistance ${r}_{F}=3.1 \, \text{m}\Omega .$ This type of coil is described in-detail in Appendix B. For resonance of the SSF the capacitance of the non-polar capacitor is ${C}_{F}=750 \, \mu \text{F}$. The AC voltage at the output of the SSF is measured using Tektronix oscilloscope model TDS 2012B as shown in Fig. 10b.

**Figure 11:**
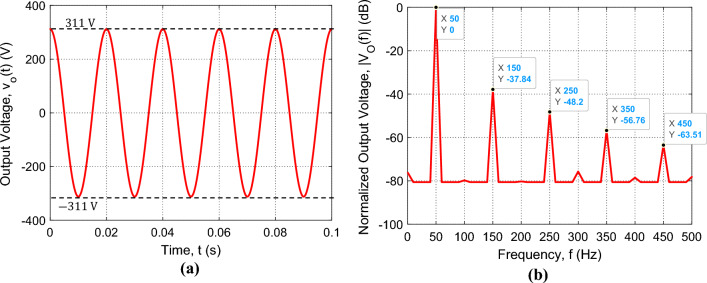
The sinusoidal voltage measured by Tektronix oscilloscope model TDS 2012B at the output of the SSF of the proposed inverter for load impedance ${Z}_{L}=55\Omega$ with periodic time $T=0.02 \, \text{s}$ and duty cycle $D=1$; the SSF parameters are ${C}_{F}=750 \, \upmu \text{F}$, ${L}_{F}=13.5 \, \text{mH}$, and ${r}_{F}=3.1 \, \text{m}\Omega$. (**a**) Time-waveform of the SSF output voltage. (**b**) The corresponding normalized spectrum magnitude, $\left|{V}_{O}\left(f\right)\right|$, obtained by the built-in FFT option of the oscilloscope.

The spectrum of the output voltage is measured through the built-in FFT option of the oscilloscope. The measured time waveform and spectrum of the voltage at the output of the SSF are presented in Fig. 11a and b, respectively, where the load impedance is ${Z}_{L}=55\Omega$ and the duty cycle is set to $D=1$. Owing to the SSF, the measured time waveform of the output voltage is almost pure sinusoidal as shown in Fig. 11a, where the amplitude ${A}_{0}$ is about 311 V ($220 \, \text{ V}$ rms). Also, Fig. 11b shows that the higher-order harmonics have very low magnitudes relative to the fundamental harmonic. This results in a very low value of the THD that is less than $-37\, \text{ dB}$ ($0.02 \%$).

### Characteristics of the inverter output without voltage regulation

The voltage at the output of the SSF can be expressed as Fourier series expansion with frequency harmonics that are multiples of ${f}_{r}$ as given by (11). As explained in Appendix A, the THD of ${v}_{O}\left(t\right)$ can be calculated as follows.

26 $${H}_{O}=\frac{1}{{{a}_{O}}_{1}}\sqrt{\sum_{n>1}{a}_{On}^{2}}$$

**Figure 12:**
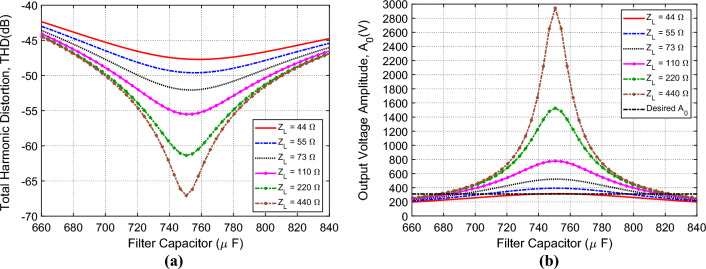
Dependence of (**a**) the THD and (**b**) the amplitude of the inverter output voltage (applied to ${Z}_{L}$) on the SSF capacitance, ${C}_{F}$, for different values of the load impedance, ${Z}_{L}$; ${L}_{F}=13.5 \, \text{mH}$; ${r}_{F}=3.1 \, \text{m}\Omega$; $D=1$.

The Proteus simulator is used to obtain the time waveform of the output voltage ${v}_{O}\left(t\right)$ for $T=0.02 \, \text{s}$ to get the fundamental harmonic ${f}_{r}=50 \, \text{Hz}$. The duty cycle is set to $D=1$, the coil inductance is ${L}_{F}=$ $13.5 \, \text{ mH}$, and the coil resistance is ${r}_{F}=1.6 \, \text{m}\Omega$. The coil is wire wound on a cylindrical core of MnZn ferrite material with ${\mu }_{r}=5000$ as described in Appendix B. The THD is then obtained by harmonic analysis using (11) and (26). Figure 12a shows the dependence of the THD on the filter capacitance ${C}_{F}$ for different values of the load impedance, ${Z}_{L}$. It is shown that the THD is decreased with increasing ${Z}_{L}$. However, the THD is below $-40 \, \text{ dB}$ ($0.01\%$) for all the indicated values of the load impedance (${Z}_{L}\ge 44\Omega$). Also, it is shown that the minimum values (local minima) of the THD is achieved at the resonance of the SSF, i.e. at ${C}_{F}={\omega }_{r}^{2}/{L}_{F}=750 \, \upmu \text{F}$. Figure 12b presents the simulation results to show the dependence of the amplitude, ${A}_{0}$, of ${v}_{O}\left(t\right)$ for ${f}_{r}=50 \, \text{Hz}$, on the filter capacitance ${C}_{F}$ for different values of the load impedance, ${Z}_{L}$. It is shown that ${A}_{0}$ increases with increasing the load impedance. Also, it is shown that the maximum values of the ${A}_{0}$ (local maxima) are achieved at the resonance of the SSF, i.e. at ${C}_{F}={\omega }_{r}^{2}/{L}_{F}=750 \, \upmu \text{F}$. It should be noted that it is required to get ${A}_{0}=311 \, \text{V}$ to get 220 V rms AC voltage at the inverter output. From the curves presented in Fig. 12, it is shown that a control system is necessary to regulate the output voltage so as to keep ${A}_{0}=311 \, \text{V}$ irrespective of the load variations. The control system proposed for voltage regulation is explained in-detail later on.

#### ${\varvec{V}}$–${\varvec{Z}}$ and ${\varvec{V}}$–${\varvec{I}}$ characteristic curves of the inverter output without voltage regulation

**Figure 13:**
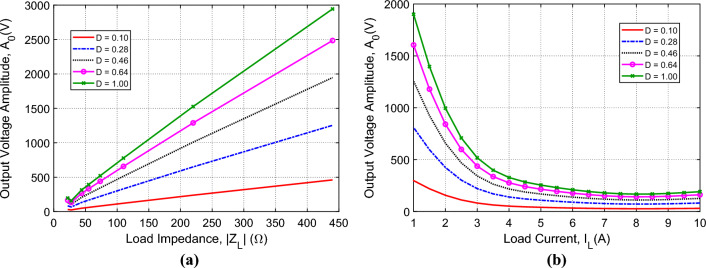
Dependence of the amplitude, ${A}_{0}$, of the output voltage, ${v}_{0}(t)$, on (**a**) the load impedance, ${Z}_{L}$, and (**b**) the load current, ${I}_{L}$, for different values of the duty cycle, $D$, without applying the voltage regulation scheme. The period $T=0.02 \, \text{s}$ (${f}_{r}=50 \, \text{Hz}$) and the SSF parameters are ${C}_{F}=750 \, \upmu \text{F}$, ${L}_{F}=13.5 \, \text{mH}$, and ${r}_{F}=3.1 \, \text{m}\Omega$.

For establishing a robust regulation mechanism of the inverter output voltage when applied to arbitrarily load impedance, it may be necessary to investigate the variation of the inverter voltage with varying the load impedance or, alternatively, with varying the load current. The ${V}_{L}$–${Z}_{L}$ characteristic curves of the inverter without voltage regulation are presented in Fig. 13a. It is shown that the load voltage decays with decreasing the load impedance. Also, for a specific value of the load current, the load voltage depends on the value of the duty cycle, $D$, defined by the expression (3). The corresponding ${V}_{L}$–${I}_{L}$ characteristic curves are presented in Fig. 13b. These curves are equivalent to the ${V}_{L}$–${Z}_{L}$ characteristic curves presented in Fig. 13. It is shown that the load voltage decays with increasing the load current. Also, for a specific value of the load current, the load voltage depends on the value of the duty cycle, $D$. From these characteristic curves, it is shown that a control system is necessary to regulate the output voltage so as to keep ${A}_{0}=311 \, \text{V}$ irrespective of the load variations. The control system proposed for voltage regulation is explained in-detail later on.

### Control system for voltage regulation, frequency stabilization, and THD Improvement

As discussed in the previous section, it has been shown that the amplitude of the load voltage ${V}_{L}$ is dependent on the load impedance, ${Z}_{L}$, or, equivalently, the load current, ${I}_{L}$. According to the load-dependent curves describing the dependence of the THD of the inverter output voltage on the duty cycle, $D$, that are presented in Fig. 13a, the value of $D$ can be determined to keep the THD below a pre-specified maximum allowable value. On the other hand, the dependence of the amplitude of the sinusoidal output on $D$ is described by the load-dependent curves presented in Fig. 13b. Thus, to stabilize the amplitude of the inverter output voltage, the value of $D$ can be changed by a regulation mechanism to compensate the change of the load impedance so as to stabilize the amplitude of the output voltage. In the meantime, it can be ensured that the selected value of $D$ satisfies the required value of the maximum allowable THD. Thus, a control system can be designed to ensure pure sinusoidal output voltage with regulated and stabilized amplitude. Moreover, this control system can stabilize the frequency by controlling the value of $T$ so as to get the output frequency exactly equal to 50 Hz.

#### Block diagram of the proposed control system

**Figure 14:**
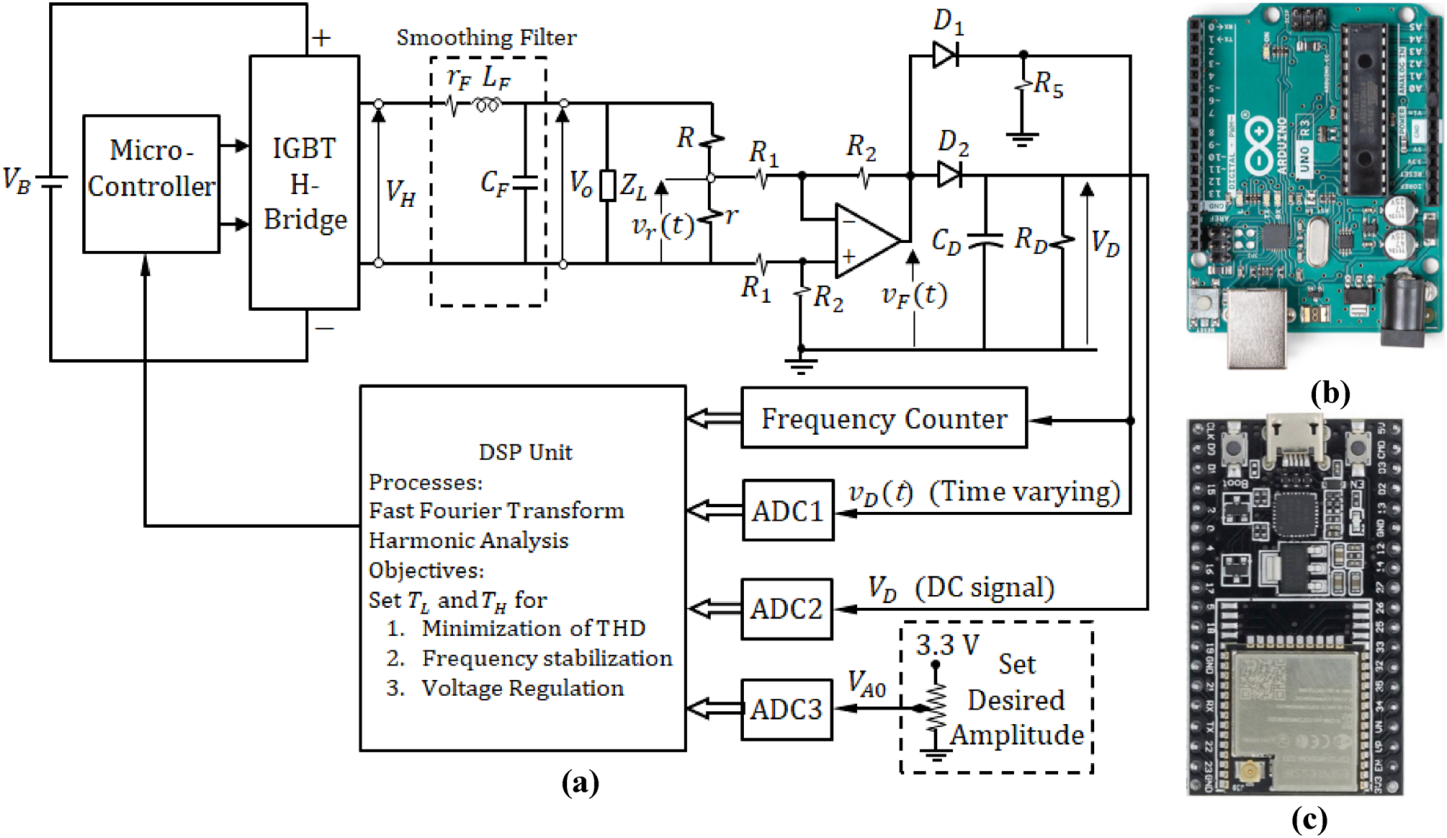
Control system for frequency stabilization, amplitude regulation and minimization of the THD of the output voltage. (**a**) Block diagram. (**b**) Arduino Uno microcontroller. (**c**) Espressif ESP32-Wroom-32 DSP unit.

The closed-loop control system shown in Fig. 14a is designed to achieve three main objectives: (i) regulation of the inverter output voltage to have a desired amplitude and stabilization of this amplitude even under impedance load variation, (ii) stabilization of the output voltage frequency at $50\text{ Hz}$ by controlling the value of $T$ (equivalently, ${T}_{L}+{T}_{H}$), and (iii) minimization of the THD of the output voltage. For fast response of the control system to the variation of the load voltage amplitude, the rectified voltage ${V}_{D}$ is used. This voltage is a DC voltage signal that is proportional to the amplitude of the sinusoidal voltage applied to the load. The frequency stabilization and the reduction of the THD require sampling of the actual time waveform of the voltage applied to the load. This can be achieved through the processing of the feedback signal ${v}_{D}(t)$. This signal is a scaled rectified version of the load voltage ${v}_{o}(t)$. Using reasonable sampling rate, the FFT of ${v}_{o}(t)$ can be obtained and, hence, the frequency of the principal harmonic can be accurately retrieved and the THD can be calculated. It should be noted that the amplitude of ${v}_{o}(t)$ can, also, be measured by the amplitude of the feedback signal ${v}_{D}(t)$. However, the measurement of the amplitude by ${V}_{D}$ is faster as there is no processing required like the case in which the amplitude of ${v}_{o}(t)$ is retrieved by processing the signal ${v}_{D}(t)$. Nevertheless, the amplitude calculated by processing the feedback signal ${v}_{D}(t)$ can be used to confirm the amplitude retrieved directly by reading ${V}_{D}$ through ADC2.

In the proposed control system, the Arduino Uno microcontroller, shown in Fig. 14b, is used for direct switching of the H-bridge, and the Espressif ESP32-wroom-32 microcontroller, shown in Fig. 14c, with the Espressif DSP library (ESP-DSP) is used to implement the DSP unit for harmonic analysis of the time waveform of the output voltage. Thus, the central unit of the control system is the ESP32 DSP whose commands are issued to the Arduino Uno microcontroller through serial communication as described by the block diagram shown in Fig. 14a.

#### Generation of the feedback signals required for the control system

The method proposed for load voltage regulation of the inverter output voltage depends on a feedback system for stabilization of the inverter output amplitude, ${A}_{o}$. The proposed feedback system is presented in Fig. 14a. A potential divider is connected in-parallel with the load impedance, ${Z}_{L}$. This potential divider is composed of a small resistance, $r$, in series with a high resistance $R$ as shown in Fig. 14a. The voltage ${V}_{r}$ is proportional to the voltage applied to the load current.

Under the assumption that $R+ r\gg \left|{Z}_{L}\right|$, one can write.

27 $${V}_{r}={V}_{o} \frac{r}{R+r}$$

The output voltage of the differential amplifier can be expressed as follows.

28 $${V}_{D}=\frac{{R}_{2}}{{R}_{1}}{V}_{r}={V}_{o} \frac{{R}_{2}r}{{R}_{1}\left(R+r\right)}$$

#### Generation of the feedback signal for frequency stabilization and minimization of THD

To facilitate harmonic analysis of the inverter output voltage for the purpose of stabilizing the frequency and minimizing the THD, the voltage signal ${v}_{D}(t)$ is generated as a scaled half-wave rectified version of ${v}_{o}(t)$. The feedback signal, ${v}_{D}(t)$, can be expressed as follows.

29 $${v}_{D}(t)=\frac{{R}_{2}r}{{R}_{1}\left(R+r\right)}{\overline{v} }_{o}(t)-{V}_{\gamma }$$

where ${\overline{v} }_{o}$ is the half-wave rectified version of ${v}_{o}(t)$.

##### Feedback signal for frequency stabilization

Measuring the frequency of the inverter output signal doesn’t require applying FFT. The control system measures the frequency of ${v}_{D}(t)$ immediately by the frequency counter hardware module shown in Fig. 14a without the need for harmonic analysis. The frequency of the inverter output voltage, ${v}_{o}(t)$, is the same as the frequency of ${v}_{D}(t)$. Thus, the output of the frequency counter module is a digital value indicating the frequency of the inverter output. This allows the DSP program to take a fast response and can immediately adjust the frequency of the inverter output voltage. This is performed by setting the correct value of $T$ and issuing this as a command to the microcontroller program.

##### Feedback signal for minimization of THD

The calculation of the THD of the inverter output voltage requires the application of harmonic analysis to the time waveform ${v}_{o}\left(t\right)$. This is simply be achieved in the DSP module by constructing this waveform from the feedback signal ${v}_{D}(t)$. The Espressif ESP32-wroom-32 microcontroller, used as the DSP module for the proposed inverter, has the ability to perform FFT and IFFT functions, required for harmonic analysis, through the Espressif DSP library^10,11^.

#### Generation of the feedback signal for amplitude regulation

Initially, the operator can set the desired amplitude of the inverter output voltage, ${v}_{o}(t)$, by setting the value of the analog input ${V}_{A0}$ using a precise, high-resolution potentiometer. This signal is fed-back to the control system (input to the DSP) through ADC3 as shown in Fig. 14a. For fast control regarding the voltage amplitude regulation, it is recommended to generate a DC signal that is directly proportional to amplitude of the inverter output voltage. For this purpose, the voltage signal ${V}_{D}$ is generated as a DC voltage proportional to the amplitude of the inverter output voltage, ${A}_{o}$, and can be expressed as follows.

30 $${V}_{D}={V}_{FM}-{V}_{\gamma }$$

where,

31 $${V}_{F}=\mathit{max}({v}_{F}\left(t\right))=\frac{{R}_{2}r}{{R}_{1}\left(R+r\right)}{A}_{o}$$

This signal can be fed-back to the voltage regulation system through ADC2, as shown in Fig. 14a, without the need for any signal processing to get fast response of voltage regulation. A comparison between the DC input voltages ${V}_{D}$ and ${V}_{A0}$ is used by the control system to regulate the amplitude of the output voltage to satisfy the requirement that ${V}_{D}={V}_{A0}$.

#### Example scenario for applying the amplitude regulation procedure

As given by (24) and explained through numerical results presented in the previous section, the amplitude,${A}_{0}$, of the inverter output voltage is strongly dependent on the magnitude of the load impedance, $\left|{Z}_{L}\right|$ and on the duty cycle, $D$, of the square-wave voltage at the output of the H-bridge. Thus, an amplitude regulation system is essentially required for the proposed voltage-source inverter to keep the amplitude constant irrespective of the load impedance. The idea of proposed amplitude regulation, proposed in the present work, is based on changing the value of $D$ to compensate the change of $\left|{Z}_{L}\right|$ so as to keep ${A}_{0}$ constant at the desired value. The feedback DC voltage signal ${V}_{D}$ can read by the DSP through ADC2 with 10-bit resolution and sampling rate of 1 MSPS. This enables the DSP to acquire the amplitude of the output voltage with high accuracy and fast rate as explained above.

**Figure 15:**
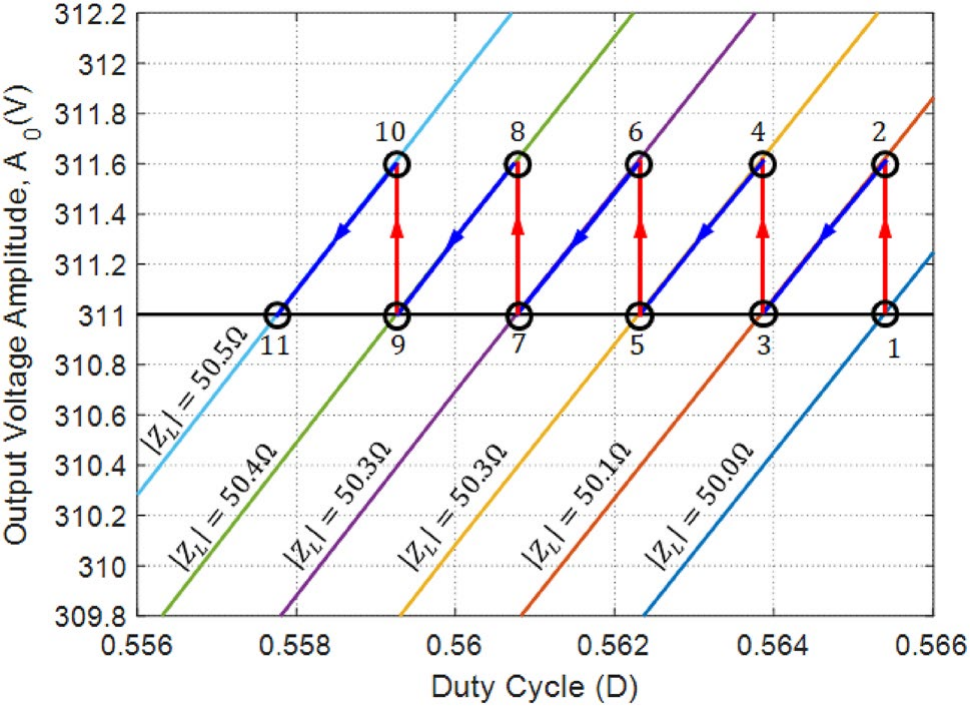
An example scenario for the amplitude regulation when the load impedance magnitude changes gradually from $50\Omega$ to $50.5\Omega$. The control system continuously changes the value of $D$ to keep the amplitude the inverter output voltage as close as possible to the desired value.

To demonstrate an example scenario of amplitude regulation, Fig. 15 presents a plot for the curves describing the dependence of ${A}_{0}$ on $D$ and for different values of $\left|{Z}_{L}\right|$. Consider the case of a sensitive load in a nuclear installation with varying impedance. It is required to energize this load with stabilized output voltage of $220 \, \text{ V}$ (i.e. ${A}_{0}=311 \, \text{V}$). Consider that the inverter is initially at the operating point 1 as shown in Fig. 15 where ${Z}_{L}=50\Omega$, $D=0.5655$, and ${A}_{0}=311 \, \text{V}$. At this point, ${Z}_{L}$ starts to gradually increase leading to increase ${A}_{0}$. Since the minimum detectable change of ${A}_{0}=0.6 \, \text{V}$ (as determined by the resolution of the ADC), the DSP can detect the change of ${A}_{0}$ when it reaches $311.6 \, \text{ V}$ and ${Z}_{L}$ reaches $50.1\Omega$. This means that the inverter is moved to operating point 2. To decrease ${A}_{0}$ back to the desired value, the DSP starts to reduce $D$ gradually (with a much faster rate than that of the load impedance change), which leads to decrease ${A}_{0}$ until the inverter reaches the operating point 3, at which ${A}_{0}=311 \, \text{V}$, $D=0.5639$, and ${Z}_{L}=50.1\Omega$. However, the load impedance continues increasing leading the move the inverter to the operating point 4 at which ${Z}_{L}=50.2\Omega$, at which the DSP detects that ${A}_{0}=311.6 \, \text{V}$. To restore ${A}_{0}$ back to the desired value, the DSP reduces the value of $D$, until the inverter reaches the operating point 5, at which $D=0.5623$, ${Z}_{L}=50.2\Omega$, and ${A}_{0}=311 \, \text{V}$. This process is repeated leading to very low level of amplitude variations around the desired value as shown in Fig. 15 that presents one of the worst case scenarios of the load impedance change. The maximum percentage amplitude fluctuation ratio ($\% {AFR}_{\text{max}}$) is the percentage ratio between the peak-to-peak voltage (PPV) of the amplitude variation to the mean value of the amplitude. During the worst-case process of voltage amplitude regulation, the $\% {AFR}_{\text{max}}$ can be evaluated as follows.

32 $${\% AFR}_{max}=\frac{rms \, of \, amplitude \, variation}{rms \, of \, the \, output \, voltage}\times 100\%=\frac{0.85}{220}\times 100\%=0.4\%$$

### Simulation and experimental assessment of the voltage-source inverter with applying the control system

The complete voltage source inverter includes the switching IGBT H-bridge, the SSF, and the control system that is constructed by the circuits generating the feedback signals, the microcontroller and the DSP unit. The programs of the Arduino Uno™ microcontroller and the Espressif ESP32-wroom-32™ microcontroller with the Espressif DSP library (ESP-DSP) are developed to perform the function of the control system as described in previous section.

#### Simulation results of the inverter output voltage under the control system

**Figure 16:**
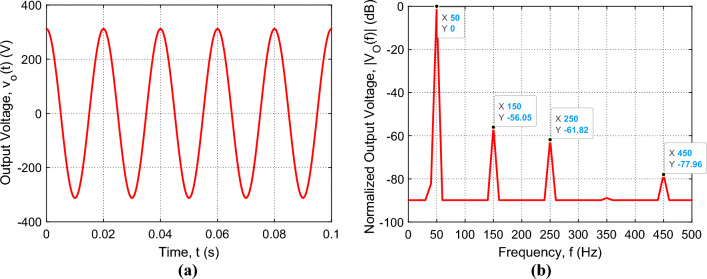
Simulation results of the sinusoidal voltage at the output of the SSF of the proposed inverter for load impedances varying from ${Z}_{L}=11\Omega$ (${I}_{L}=20 \, \text{A}$) to ${Z}_{L}=440\Omega$ (${I}_{L}=0.5 \, \text{A}$) with periodic time $T=0.02 \, \text{s}$; the SSF parameters are ${C}_{F}=750 \, \upmu \text{F}$, ${L}_{F}=13.5 \, \text{mH}$, and ${r}_{F}=3.1 \, \text{m}\Omega$. (**a**) Time-waveform of the inverter output voltage, ${v}_{O}(t)$. (**b**) The corresponding normalized spectrum magnitude, $\left|{V}_{O}\left(f\right)\right|$, obtained by FFT.

The Proteus simulator is used to simulate the complete design of the proposed voltage source inverter that is presented in Fig. 14a. The entire system including the programs of the Arduino Uno and the ESP32-wroom-32 microcontrollers is subjected to simulation for examining the inverter operation. When the operation of the complete system including the IGBT H-bridge, the SSF, the feedback circuit, the DSP unit, and the microcontroller is simulated in the Proteus simulator, the time waveform and the spectrum of the inverter output voltage are presented in Fig. 16, where the load impedance varies from ${Z}_{L}=11\Omega$ (${I}_{L}=20 \, \text{A}$) to ${Z}_{L}=440\Omega$ (${I}_{L}=0.5 \, \text{A}$). To get the amplitude, ${A}_{0}$ stabilized at $311 \, \text{V}$ ($220 \, \text{ V \, rms}$), the DSP varies the duty cycle $D$ to compensate the change of ${A}_{0}$ due to the variation of ${Z}_{L}$ as described before. Owing to the application of the control procedure described in the previous section, the time waveform of the inverter voltage is pure sinusoidal as shown in Fig. 16a. It is shown that the amplitude ${A}_{0}$ is stable at, exactly, $311 \, \text{V}$ for load impedances varying from $11\Omega$ to $440\Omega$. Also, it is shown in Fig. 16b that the higher-order harmonics are depressed as they have very low magnitudes relative to the fundamental harmonic. This results in a very low value of the THD that is less than $-57 \, \text{ dB}$ ($0.0002 \%$).

#### Experimental measurement of the inverter output voltage under the control system

**Figure 17:**
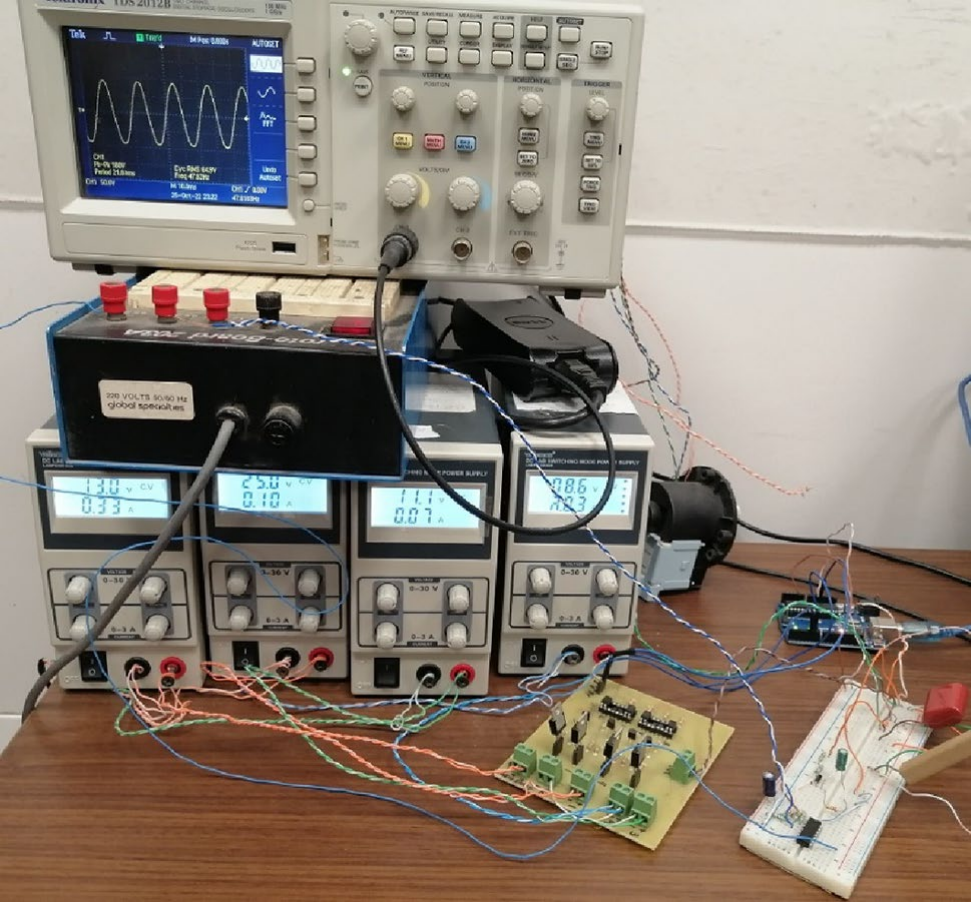
Experimental testing of the fabricated prototype of the complete voltage-source inverter including the control system described in the previous section.

The complete circuits of the inverter with the proposed control system are fabricated and subjected to experimental measurements. The fabricated prototype of the voltage-source inverter includes the IGBT H-bridge, the sinusoidal shaping SSF, the Arduino Uno™ microcontroller, and the Espressif ESP32-wroom-32 microcontroller with the Espressif DSP library (ESP-DSP). The experimental measurements are performed by the Tektronix oscilloscope model TDS 2012B as shown in Fig. 17. The built-in FFT facility of the oscilloscope is used to measure the spectrum of the output voltage.

**Figure 18:**
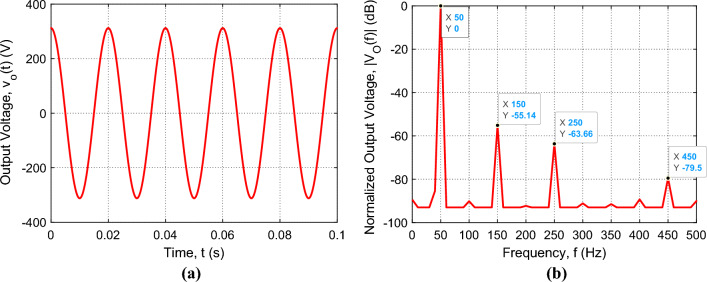
The sinusoidal voltage measured by Tektronix oscilloscope model TDS 2012B at the output of the SSF of the proposed inverter for load impedances varying from ${Z}_{L}=11\Omega$ (${I}_{L}=20 \, \text{A}$) to ${Z}_{L}=440 \Omega$ (${I}_{L}=0.5 \, \text{A}$) with periodic time $T=0.02 \, \text{s}$; the SSF parameters are ${C}_{F}=750 \, \upmu \text{F}$, ${L}_{F}=13.5 \, \text{mH}$, and ${r}_{F}=3.1 \, \text{m}\Omega$. (**a**) Time-waveform of the inverter output voltage, ${v}_{O}(t)$. (**b**) The corresponding normalized spectrum magnitude, $\left|{V}_{O}\left(f\right)\right|$, obtained by the FFT option of the oscilloscope.

The measured time waveform and the corresponding spectrum of the inverter output voltage are presented in Fig. 18a and b, respectively, for load impedances varying from ${Z}_{L}=11\Omega$ (${I}_{L}=20 \, \text{A}$) to ${Z}_{L}=440\Omega$ (${I}_{L}=0.5 \, \text{A}$). Owing to the application of the control procedure described in the previous section, the measured time waveform of the inverter voltage is pure sinusoidal as shown in Fig. 18a, where the amplitude ${A}_{0}$ is shown to be stable at, exactly, $311 \, \text{V}$. Also, Fig. 18b shows that the higher-order harmonics are depressed as they have very low magnitudes relative to the fundamental harmonic. This results in a very low value of the THD that is less than $-57 \, \text{ dB}$ ($0.00025 \%$). Comparing the results shown in Fig. 16 to those shown in Fig. 18, it becomes clear that the simulation results and experimental measurements of the proposed voltage source inverter agree well with each other.

### Power loss and efficiency of the voltage-source inverter

#### Power loss in the voltage-source inverter

There are four main causes of power loss in the proposed inverter design; these can be summarized as follows.

- Power dissipation due to switching losses in the H-bridge, ${P}_{S}$.
- Heat power dissipation in the IGBTs of the H-bridge, ${P}_{H}$.
- Power dissipation in the control system, ${P}_{C}$.
- Power dissipation in the internal resistance, ${r}_{F}$, of the coil of SSF, ${P}_{F}$.

Thus, the total power loss can be expressed as follows.

33 $${P}_{L}= {P}_{S}+{P}_{H}+{P}_{C}+{P}_{F}$$

#### IGBT switching loss

In one time period (cycle) of the inverter output voltage, each of the four IGBT is switched “ON” and “OFF” once. Let the total switching energy for a single IGBT be ${\text{e}}$. Thus, the total switching loss of the inverter can be expressed as follows.

34 $${P}_{S}=8{\text{e}}{f}_{r}$$

where, ${f}_{r}$ is the frequency of the inverter output voltage (consider ${f}_{r}=50 \, \text{ Hz}$ for the present case).

The ON/OFF switching losses (for one IGBT in one cycle of the output voltage) are given in the datasheet^12^ for IC of 15 A and VCC of 400 V. Thus, one can set ${\text{e}}=0.7 \, \text{mJ}$ in (34) as a typical value of the switching loss.

35 $${P}_{S}=8\times 0.7\times {10}^{-3}\times 50=0.28 W, {I}_{L}=15 \, \text{A}$$

Thus, the switching power loss is very small and can be negligible. However, for load current ${I}_{L}=10 \, \text{A}$, the switching loss, ${P}_{S}$, doesn’t exceed the value given by (35).

#### Heat power dissipation

**Figure 19:**
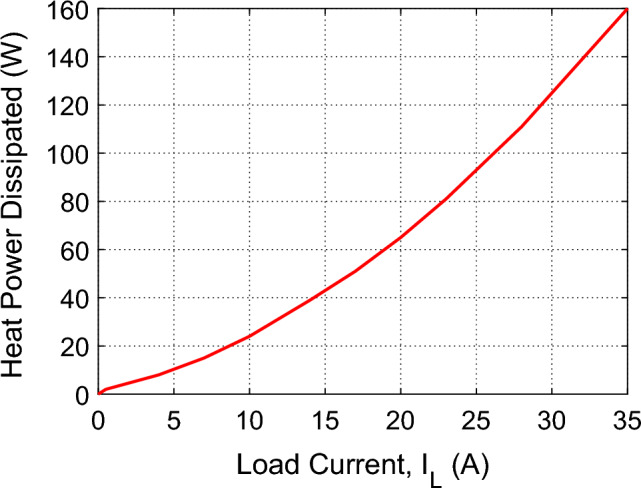
Heat power dissipated in one IGBT of the H-bridge as a function of the load current^12^.

The heat power dissipated in one IGBT (model Infineon SGP15N60) of the H-bridge is presented in Fig. 19 as a function of the load current as given in the manufacturer datasheet^12^. It is shown that the dissipated power in the form of heat energy exhibits a second-order dependence on the IGBT collector current. For the IGBT H-Bridge presented in Fig. 4, the load current is approximately equal to the IGBT collector current. Since, the proposed voltage-source inverter is designed for loads that are, typically, limited to 10 A current, from the curve presented in Fig. 19, one can write,

36 $${P}_{H}=2\times 25=50 W, {I}_{L}=10 \, \text{A}$$

#### Power dissipation in the control circuit

The power dissipated in the circuits of the control system shown in Fig. 15 is the sum of the power dissipated in the Arduino Uno microcontroller, the power dissipated in the Espressif ESP32-Wroom-32 microcontroller, and the power dissipated in the feedback circuit. Due to the high input impedance of the OP-Amp-based differential amplifier, the power dissipated in the control circuit can be approximated as follows.

37 $${P}_{C}={P}_{\mu }+{P}_{DSP}+\frac{{\left|{V}_{0}\right|}^{2}}{r+R} \approx {P}_{\mu }+{P}_{DSP}+ \frac{{\left|{V}_{0}\right|}^{2}}{R}$$

where, ${P}_{\mu }$ is power dissipated in the microcontroller (typically $1.07 \, \text{ W}$), ${P}_{DSP}$ is the power dissipated in the DSP unit and the three ADCs (typically $0.7 \, \text{ W}$). Thus, for a resistive load of ${Z}_{L}=22\Omega$ (i.e. ${I}_{L}=10 \, \text{A}$), and considering that $r=5.33 \, \text{k}\Omega$ and $R=1 \, \text{M}\Omega$, the power dissipated in the control unit can be calculated as follows.

38 $${P}_{C}\approx 1.07+ 0.7+{10}^{2} \times \frac{{22}^{2}}{{10}^{6}}=1.8 W, {I}_{L}=10 A$$

Thus, the power dissipated in the control system is much smaller than the power delivered to the load.

#### Power dissipation in the SSF

According to^13^, the magnetic loss of the core made of MnZn ferrite N87 (the material used for the coil used in the proposed inverter design) is negligible for frequencies below 1 kHz. Therefore, the magnetic loss of the ferrite core is neglected in the present study. Thus, the power dissipated in the SSF can be approximated as follows.

39 $${P}_{F}\approx {I}_{L}^{2} {r}_{F}$$

A coil of high-quality wire has a small ${r}_{F}$ and, hence, the dissipated power in very low in comparison to the power delivered to the load. In Appendix B^14,15^ it is shown that a coil of MnZn ferrite core with ${D}_{C}=3 \, \text{cm}$, ${D}_{W}=2 \, \text{mm}$, and $N=6$ has inductance $L=13.5 \, \text{mH}$ and internal resistance ${r}_{F}=1.6 \, \text{m}\Omega$. In this case, for a load current ${I}_{L}=10 \, \text{A}$,

40 $${P}_{F}\approx {10}^{2}\times 0.0031=0.31 W, {I}_{L}=10 \, \text{A}$$

**Figure 20:**
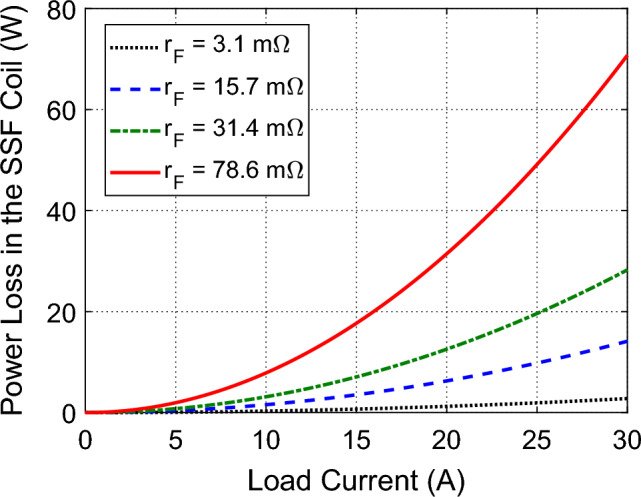
Power dissipated in the SSF as a function of the load current, ${I}_{L}$ for different values of the coil resistance, ${r}_{F}$.

For the same value of the coil inductance (${L}_{F}=13.5 \, \text{mH}$) used for optimum design of the proposed voltage-source inverter, the coil resistance, ${r}_{F}$, depends on the relative permeability, ${\mu }_{r}$, of the ferrite core as shown in Table B.2 of Appendix B. The dependence of the power dissipated in the coil of the SSF on the load current, ${I}_{L}$, for different values of the coil resistance, ${r}_{F}$, is presented in Fig. 20. It is shown that using a ferrite core of higher permeability results in a lower value of ${r}_{F}$ and, consequently, reduces the power loss in the coil of the SSF.

#### Total power dissipation

By summation of the four types of power loss encountered in the proposed voltage-source inverter as explained above, the total power loss can be evaluated. Table 2 gives a summary of the typical values of the power dissipated in the proposed voltage-source inverter for output voltage ${V}_{0}=220 \, \text{V}$ and load current ${I}_{L}=10 \, \text{A}$, where the SSF design parameters are set to ${C}_{F}=750 \, \upmu \text{F}$, ${L}_{F}=13.5 \, \text{mH}$, and ${r}_{F}=3.1 \, \text{m}\Omega$.

**Table 2:** Typical values of the power dissipated in the proposed voltage-source inverter for load current ${I}_{L}=10 \, {\text{A}}$ (i.e. ${Z}_{L}=22 \Omega$).

Type of power loss	Value of power loss (W)
${P}_{S}$	0.28
${P}_{H}$	50.00
${P}_{C}$	1.80
${P}_{F}$	0.31
Total power loss ($${P}_{L})$$	52.39

The SSF design parameters are ${C}_{F}=750 \, \upmu \text{F}$, ${L}_{F}=13.5 \, \text{mH}$, and ${r}_{F}=3.1 \, \text{m}\Omega$.

### Efficiency of the voltage-source inverter

The power, ${P}_{o}$, delivered to the load is calculate as follows.

40 $${P}_{o}={I}_{L}^{2} {R}_{L}$$

Thus, the typical efficiency of the proposed voltage-source inverter for ${V}_{0}=220 \, \text{V}$, ${I}_{L}=10 \, \text{A}$, and for the total power loss as evaluated in the previous sub section can be obtained as follows.

41 $$\eta = \frac{{P}_{o}}{{P}_{o}+{P}_{L}}=\frac{2200}{2200+52.39}\times 100 \%=97.7 \%$$

**Figure 21:**
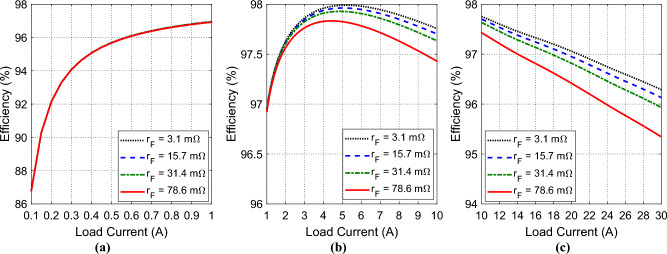
Efficiency of the proposed voltage-source inverter as a function of the load current in the range (**a**) ${I}_{L}=0.1 \text{to }1.0 \, \text{A}$, (**b**) ${I}_{L}=1.0 \text{to }10 \, \text{A}$, and (**c**) ${I}_{L}=10 \text{to }30 \, \text{A}$, for different values of the coil resistance, ${r}_{F}$.

However, the efficiency of the proposed inverter is plotted versus the load current, ${I}_{L}$, for different values of the coil resistance, ${r}_{F}$, as shown in Fig. 21. For low values of the load current, $0.1\le {I}_{L}\le 1.0 \, \text{A}$, the efficiency monotonically increases from $87\%$ to $97\%$ as shown in Fig. 21a. It is clear that, for such low values of the load current, the inverter efficiency is almost independent of the coil resistance, ${r}_{F}$. When the load current lies within the range, $1.0\le {I}_{L}\le 10 \, \text{A}$, the efficiency ranges between $97\%$ and $98\%$ with a peak value of $98\%$ as shown in Fig. 21b. For large values of the load current, $10\le {I}_{L}\le 30 \, \text{A}$, the efficiency monotonically decreases from about $97.5\%$ to about $95.5\%$ shown linear dependence as shown in Fig. 21c. It is clear that for ${I}_{L}>1 \, \text{A}$, the inverter efficiency decreases with increasing ${r}_{F}$.

### Comparison with other inverter designs

A summary of comparisons with other designs of voltage-source inverters available in literature is presented in Table 3. Note that the performance measures THD, AE, AFR, FE, FFR, and $\eta$ are defined above in the third section titled “Stability requirements of the voltage source”. The list of comparison includes six performance parameters: the AE and AFR as measures of amplitude stability, the FE and FFR as measures of frequency stability, the THD (dB) as a measure of sinusoidal shape purity of the output voltage, and the inverter efficiency. It is shown that the voltage-source inverter proposed in the present work gives the lowest THD and the highest efficiency among the other inverter designs listed in Table 2. Also, the performance measures, AE, AFR, FE, and FFR are not mentioned explicitly in the listed publications. It is shown in Table 3 that the design requirements listed in Table 1 are achieved by the proposed design of the voltage-source inverter.

**Table 3:** Performance comparison with other voltage-source inverter designs available in literature.

Work	THD (dB)	AE (%)	AFR (%)	FE (%)	FFR (%)	$\eta$ (%)
^7^	$-27.1$	NA	NA	NA	NA	95.2
^16^	$-26.0$	NA	NA	NA	NA	93.8
^17^	$-25.0$	NA	NA	NA	NA	94.1
^18^	$-24.8$	NA	NA	NA	NA	96.7
^19^	$-22.0$	NA	NA	NA	NA	93.7
^20^	$-25.0$	NA	NA	NA	NA	94.1
^21^	$-28.0$	NA	NA	NA	NA	90.0
Present	$-57.0$	0.5	0.2	0.3	0.15	97.7

## Discussion

A novel design of a stabilized single-phase voltage-source inverter has been designed to be used in photovoltaic systems employed in nuclear installations for feeding sensitive loads. Such types of loads require a voltage source with quite stabilized magnitude and frequency. The inverter is based on an H-bridge of four type IGBTs with gate drivers and optocoupler isolators. The frequency and magnitude stabilization has been achieved through a robust control system where the Arduino Uno microcontroller is used for direct switching of the H-bridge and the Espressif ESP32-wroom-32 microcontroller with the Espressif DSP library (ESP-DSP) is used to implement the DSP unit for harmonic analysis of the time waveform of the output voltage. The central unit of the control system is the ESP32 DSP whose commands are issued to the Arduino Uno microcontroller through serial communication. A feedback system has been designed to measure the frequency and amplitude of the output voltage applied to the load. The frequency measurement is achieved through a frequency counter whereas the amplitude measurement is carried out through a fast ADC. It is shown that proposed control system is able to stabilize both the amplitude and frequency of the voltage applied to the load and, also, reduces the switching losses of the IGBTs of the inverter. The Proteus simulator is used to investigate the performance of the proposed voltage-source inverter. Also, a prototype has been fabricated for the proposed inverter for experimental evaluation of its performance. Both the simulation results and the experimental measurements have shown that the proposed inverter has stabilized magnitude and frequency with almost zero THD. Also, the inverter efficiency has been shown to be greater than 97%.

### Supplementary Information

Supplementary Information.

## Data Availability

All data generated during this study are included in this article.
