# Supplementary material for: Stabilized voltage source inverter for sensitive loads in nuclear installations
Source: Sci Rep. 2024 Jul 4;14:15419. doi: 10.1038/s41598-024-65331-7 (PMC11224377; doi:10.1038/s41598-024-65331-7)
Supplement: Supplementary file 1 — Supplementary Information. [file 41598_2024_65331_MOESM1_ESM.docx]

# Appendix A

# Harmonic distortion analysis

Harmonic distortion in the electric power systems means the existence of (significant) undesired frequency harmonics in the power source supplied by the power system. These unwanted components are integer multiples of the principal (desired) frequency and may degrade the performance of the power system. The desired frequency is usually 50 or 60 Hz. One of the most common metrics used for quantitative evaluation of the harmonic distortion is the total harmonic distortion (THD), which measures the distortion of the voltage or current time waveform in comparison to a pure sinusoidal waveform of the desired frequency. It is a global ratio that is evaluated as the ratio the sum of the power of all the undesired harmonics to the power of the fundamental harmonic. The THD is usually expressed as a percentage or decibel (dB). For very low THD, it is preferred to be expressed in dB. High THD in a power system indicates high level of distortion, which can negatively affect the electric equipment existing in the power system. To evaluate the THD of the voltage source, the output voltage can be expressed in a Fourier series as follows.

| $v\left( t \right)=\sum_{n=1}^{\infty} a_{n}\cos(\omega_{n}t)=\sum_{n=1}^{\infty} a_{n}\cos\left( n\omega_{r}t \right)$ | (A.1) |
| --- | --- |

where, $\omega_{r}=2\pi f_{r}$ and $f_{r}$ is the desired or principal frequency.

Let $H$ be the THD of the output voltage; this can be expressed as follows.

| $H=\frac{1}{a_{1}}\sqrt{\sum_{n\geq2} a_{n}^{2}}$ | (A.2) |
| --- | --- |

where $a_{n}$ is the magnitude of the $n^{\mathrm{th}}$-order harmonic of the Fourier expansion. As the desired frequency, $f_{r}$, of output voltage is usually the dominant frequency harmonic of the time waveform, i.e. $f_{r}=f_{1}$, the magnitude of the $1^{\mathrm{st}}$-order harmonic, $a_{1}$, is usually much greater than the higher-order harmonics (i.e. $a_{1}\gg a_{n}, n\geq2$). The root-mean-squared (rms) value of the output voltage, $V_{rms}$, can be expressed as follows.

| $V_{rms}=\sqrt{\sum_{n=1}^{\infty} a_{n}^{2}}$ | (A.3) |
| --- | --- |

In terms of the principal harmonic magnitude, $a_{1}$, and the THD, $V_{rms}$ can be expressed as follows.

| $V_{rms}=a_{1}\sqrt{1+H^{2}}$ | (A.4) |
| --- | --- |

# Appendix B

# Wire-Wound Coil with MnZn Ferrite Core

A coil can be made by winding a wire on a cylindrical core as shown in Figure A.1. This coil is required to be of low profile and to give high inductance and low internal resistance.


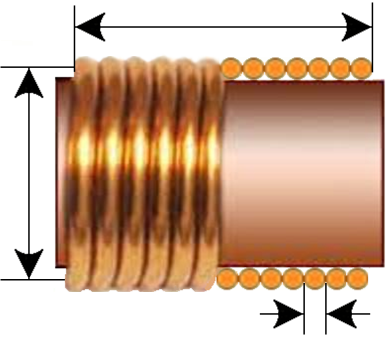

$$L_{C}$$

$$D_{C}$$

$$D_{W}$$

**Figure B.1:** Wire-wound coil made a copper wire wrapped on a cylindrical core.

## Calculation of the Wire-Wound Inductance

The inductance of the wire-wound coil can be calculated using the following formula.

| $L_{F}=\frac{\pi\mu_{r}\mu_{0}N^{2}D_{C}^{2}}{4L_{C}}$ | (B.1) |
| --- | --- |

where $\mu_{r}$ is the relative permeability of the core material, $\mu_{0}$ is the permeability of free space, $N$ is the number of turns, $D_{C}$ is the diameter of the coil, and $L_{C}$ is length of the coil.

Considering that there is separation between the turns of the coil except for the negligible insulator thickness, one can consider that $L_{C}=ND_{W}$. Thus, the expression (B.1) can be written as follows.

| $L_{F}= \frac{\pi N\mu_{r}\mu_{0}D_{C}^{2}}{4D_{W}}$ | (B.2) |
| --- | --- |

The internal resistance of the coil can be calculated using the following formula.

| $r_{F}= \rho\frac{L_{W}}{A_{W}}$ | (B.3) |
| --- | --- |

where $\rho$ is the electric resistivity of the wire material, $L_{W}=\pi D_{C}N$ is the wire length, $A_{W}=\pi D_{W}^{2}/4$ is the area of the wire cross section.

The internal resistance $r_{F}$ can be expressed as follows.

| $r_{F}= 4N \frac{\rho D_{C}}{D_{W}^{2}}$ | (B.4) |
| --- | --- |

Given that a certain value of the inductance, $L_{F}$, is required, the number of turns can be calculated as follows.

| $N=\frac{2}{D_{C}}\sqrt{\frac{L_{F}L_{C}}{\pi\mu_{r}\mu_{0}}}$ | (B.5) |
| --- | --- |

Alternatively, the number of turns, $N$, can be expressed in terms of the coil inductance as follows.

| $N=\frac{4D_{W}}{\pi\mu_{r}\mu_{0}D_{C}^{2}} L_{F}$ | (B.6) |
| --- | --- |

Also, for a given value of $L_{F}$, the coil resistance, $r_{F}$ can be calculated as follows.

| $r_{F}= \alpha L_{F}$ | (B.7) |
| --- | --- |

where, $\alpha$ is a factor that depends on the wire and core material and diameters and is expressed as follows.

| $\alpha= \frac{16 \rho}{\pi\mu_{r}\mu_{0}D_{C}D_{W}}$ | (B.8) |
| --- | --- |

Assuming there no spacing between the coil turns, the coil length, $L_{C}$, can be calculated for a given value of $L$ as follows.

| $L_{C}=ND_{W}=\frac{4D_{W}^{2}}{\pi\mu_{r}\mu_{0}D_{C}^{2}} L_{F}$ | (B.9) |
| --- | --- |

## Cylindrical Coil with (MnZn) Ferrite Core

The manganese zinc (MnZn) ferrite cores are characterized by high permeability, high relative permittivity and high resistivity [20]. The relative permeability lies within the range $750 -2\times{10}^{5}$ [20]. Also, high magnetic permeability in combination with low electrical conductivity helps to prevent eddy currents in the ferrite cores. This enables the MnZn ferrite cores to have the advantage of low eddy currents (i.e. low core loss) at different frequencies. For example, to produce a coil of inductance $L_{F}=13.5 \mathrm{mH}$, the optimum design parameters are listed in Table B.1.

| Parameter | $D_{w}$ | $D_{C}$ | $L_{c}$ | $N$ | $\mu_{r}$ | $\rho$ |
| --- | --- | --- | --- | --- | --- | --- |
| Value | $2 mm$ | $3 cm$ | $1.2 cm$ | $6$ | $5000$ | $1.724\times{10}^{-8} \Omega.m$ |

**Table B.1:** Typical values of the wire-wound coil on MnZn ferrite core to achieve coil inductance $L_{F}=13.5 \mathrm{mH}$ and coil resistance $r_{F}=3.1 m\Omega$.

The application of equations (B.1), (B.8), and (B.7) leads to the following values of the remaining coil parameters.

$$L_{F}=\frac{\pi\times6{\times5000\times4 \pi\times10}^{-7}\times{0.03}^{2}}{4\times0.002}= 13.5 mH$$

$$\alpha=\frac{16\times1.724\times{10}^{-8}}{\pi{\times5000\times4 \pi\times10}^{-7}\times0.03\times0.002}= 0.233 \Omega.H^{-1}$$

$$r_{F}=3.1$$

## Effect of the MnZn ferrite core permeability on the coil resistance and dimensions

To design a coil of desired inductance ($L_{F}=13.5 \mathrm{mH}$ for the present case, the ferrite core permeability has a significant effect on the size and resistance of the coil. Table B.2 gives a list of some possible values of the relative permeability, $\mu_{r}$, and the corresponding values of the coil resistance, $r_{F}$, length, $L_{C}$, and number of turns, $N$.

| $\mu_{r}$ | $r_{F} (m\Omega)$ | $L_{C} (cm)$ | $N$ |
| --- | --- | --- | --- |
| $5000$ | $3.1$ | $1.2$ | $6$ |
| $4000$ | $3.9$ | $1.5$ | $8$ |
| $2000$ | $5.2$ | $2.0$ | $10$ |
| $2000$ | $7.9$ | $3.0$ | $15$ |
| $1000$ | $15.7$ | $6.0$ | $30$ |
| $500$ | $31.4$ | $12.2$ | $61$ |

**Table B.2:** Design parameters of wire-wound coil on MnZn ferrite core to produce coil inductance $L_{F}=13.5 \mathrm{mH}$; the other design parameters of the coil are $D_{W}=2 \mathrm{mm}$ and $D_{C}=3 \mathrm{cm}$.

The MnZn ferrite core of the SSF coil is fabricated using the compressed powder technique to get relatively high value of the saturation magnetic flux density, $B_{s}=650 m$, which is very close to that obtained in [21]. Table B.3 gives a list of the optimum design parameters of the wire-wound coil on MnZn ferrite core to produce coil inductance $L_{F}=13.5 \mathrm{mH}$ without magnetic saturation of the MnZn core for output currents up to $10 A$ ($2.2 kW$ output power).

| $\mu_{r}$ | $D_{W}(mm)$ | $D_{C}(cm)$ | $L_{C} (cm)$ | $r_{F} (m\Omega)$ | $N$ |
| --- | --- | --- | --- | --- | --- |
| $500$ | $1.4$ | $7.2$ | $1.0$ | $18.7$ | $7$ |

**Table B.3:** Optimum design parameters of the wire-wound coil on MnZn ferrite core to produce coil inductance $L_{F}=13.5 \mathrm{mH}$ without magnetic saturation of the MnZn core for output currents up to $10 A$ ($2.2 kW$ maximum output power).
